# Are the literacy difficulties that characterize developmental dyslexia associated with a failure to integrate letters and speech sounds?

**DOI:** 10.1111/desc.12423

**Published:** 2016-08-06

**Authors:** Hannah M. Nash, Debbie Gooch, Charles Hulme, Yatin Mahajan, Genevieve McArthur, Kurt Steinmetzger, Margaret J. Snowling

**Affiliations:** ^1^ Department of PsychologyUniversity of LeedsUK; ^2^ Division of Psychology and Language SciencesUniversity CollegeLondonUK; ^3^ The MARCS InstituteUniversity of Western SydneyAustralia; ^4^ ARC Centre of Excellence in Cognition and its DisordersMacquarie UniversityAustralia; ^5^ St John's CollegeUniversity of OxfordUK

## Abstract

The ‘automatic letter‐sound integration hypothesis’ (Blomert, 2011) proposes that dyslexia results from a failure to fully integrate letters and speech sounds into automated audio‐visual objects. We tested this hypothesis in a sample of English‐speaking children with dyslexic difficulties (*N *=* *13) and samples of chronological‐age‐matched (CA; N = 17) and reading‐age‐matched controls (RA;* N *=* *17) aged 7–13 years. Each child took part in two priming experiments in which speech sounds were preceded by congruent visual letters (congruent condition) or Greek letters (baseline). In a behavioural experiment, responses to speech sounds in the two conditions were compared using reaction times. These data revealed faster reaction times in the congruent condition in all three groups. In a second electrophysiological experiment, responses to speech sounds in the two conditions were compared using event‐related potentials (ERPs). These data revealed a significant effect of congruency on (1) the P1 ERP over left frontal electrodes in the CA group and over fronto‐central electrodes in the dyslexic group and (2) the P2 ERP in the dyslexic and RA control groups. These findings suggest that our sample of English‐speaking children with dyslexic difficulties demonstrate a degree of letter‐sound integration that is appropriate for their reading level, which challenges the letter‐sound integration hypothesis.

## Research highlights

- According to the novel ‘letter‐sound integration hypothesis’, dyslexia is caused by a failure to fully integrate letters and speech sounds into audio‐visual objects. We tested this hypothesis using a priming task to measure behavioural and brain responses to letter‐primed speech sounds in children with dyslexic difficulties and two groups of controls (age matched and reading‐age matched).
- We found evidence to suggest that there is a developmental shift from letter‐sound associations to automatic integration in children learning to read English that is driven by reading experience.
- Our sample of children with dyslexic difficulties showed similarities with both age‐matched and reading‐age‐matched controls. This suggests that they have developed a degree of letter‐sound integration that is in line with their reading ability, which presents a challenge to the letter‐sound integration hypothesis.

## Introduction

Dyslexia is a difficulty in learning to read that affects 3–8% of children (Peterson & Pennington, 2012). The dominant causal theory posits that it arises from a phonological impairment (Hulme & Snowling, 2009). However, according to a recent alternative hypothesis, the cause of dyslexia is a failure to adequately integrate letters and speech sounds into fully automated audio‐visual objects, which in turn arises from a neural cross‐modal binding deficit (Blomert, 2011). The ‘letter‐sound integration’ (LSI) hypothesis distinguishes between simply knowing letter sounds, and the development of automatic associations. Such automatic associations enable the efficient activation of speech sounds from letters that are essential for decoding. Thus, according to the LSI hypothesis, it is a lack of automatization that causes the decoding problems that characterize dyslexia. The purpose of the current study was to test the validity of the LSI hypothesis in English‐speaking children with dyslexia.

Letter‐sound integration has been studied both behaviourally and using neurophysiological techniques. An early behavioural study found that Dutch adults were significantly faster to identify the vowel in an auditorily presented syllable (e.g. /a/ in /ka/) when it had been primed by a co‐occurring, congruent, visually presented letter (i.e. ‘a’) (Dijkstra, Schreuder & Frauenfelder, 1989). Priming was greatest when the letter preceded the vowel by either 250 ms or 100 ms. The authors concluded that this priming reflects automatic cross‐modal activation of the speech sound from the letter and that this process supports visual word recognition.

In a more recent behavioural study, Blau, Reithler, van Atteveldt, Seitz, Gerretsen *et al*. (2010) asked children with dyslexia and typically developing (TD) children to respond ‘same or different’ to simultaneously visually presented letters and speech sounds. On half of the trials, the two stimuli were congruent (‘t’ /t/; i.e. ‘same’) while on the other half they were incongruent (‘n’ /t/; i.e. ‘different’). Although children with dyslexia were as accurate as TD children, they were significantly slower, which the authors interpreted as evidence for reduced automaticity of LSI. However, there was no baseline condition or control for general response speed. Thus, this group effect may reflect the fact that children with dyslexia were slower overall.

In an early neurophysiological study of LSI using magnetoencephalography (MEG), Raij, Uutela and Hari (2000) recorded neuromagnetic cortical responses while Finnish typical adult readers completed a monitoring task in which auditory (speech sounds), visual (letters) or audio‐visual (matching or non‐matching) stimuli were presented. They found that presenting matching letters and sounds led to a suppressive effect at 380–450 ms. A reduced suppressive effect was observed for non‐matching letters. They concluded that bilateral superior temporal sulci (STS) regions are multisensory areas that integrate letters and sounds. In their behavioural data, RTs were faster to audio‐visual stimuli.

Subsequent evidence for the LSI hypothesis comes from functional magnetic resonance imaging (fMRI) studies that have compared Dutch‐speaking adults and children with dyslexia to age‐matched controls. Van Atteveldt, Formisano, Goebel and Blomert (2004) isolated brain regions involved in the processing of letters and speech sounds in typical adult readers. Bilateral STS and superior temporal gyrus (STG) regions were activated by both letters and speech sounds in isolation (heteromodal areas), but were more activated when these were presented together (bimodal enhancement). Furthermore, the anterior region of the STS responded only to bimodal presentation, highlighting this as a putative binding site. Crucially, they also showed that the presentation of a congruent letter‐speech sound pair resulted in an increase in activation in the primary auditory cortex, Heschl's sulcus (HS) and the planum temporale (PT) compared to activation for speech sounds in isolation (consistent across participants in the left hemisphere). This suggested that visual letters influenced the processing of speech sounds, which was attributed to feedback from the STS and STG (heteromodal and bimodal areas) to the primary auditory cortex.

In a later fMRI study, Blau, van Atteveldt, Ekkebus, Goebel and Blomert (2009) investigated the neural integration of letters and speech sounds in adults with dyslexia. Participants passively attended to isolated letters and speech sounds, as well as letter‐speech sound pairs that were either congruent (e.g.’t’ /t/) or incongruent (e.g. ‘n’ /t/). The typical readers showed significantly greater activation for congruent than incongruent pairs, that is, a ‘congruency effect’. The adults with dyslexia did not show this significant difference in activation. However, a limitation of this finding is that the congruency effect in typical readers could have arisen from an increase in activation in the congruent condition or a decrease in activation in the incongruent condition. In fact, the key comparison is between activation for speech sounds presented with visual letters (arguably reflecting the reading process) and activation to speech sounds presented in isolation. In Blau *et al*.'s (2009) study, typical adult readers showed a non‐significant increase in activation for the congruent letter‐sound pairs compared to activation for isolated speech sounds in the STG, as well as a significant reduction in activation for incongruent pairs compared to speech sounds. Similarly, the adults with dyslexia showed a non‐significant increase in activation in the congruent condition compared to isolated speech sounds, which could indicate that LSI is operating normally. In contrast, they did not show reduced activation for incongruent letter‐sound pairs compared to isolated speech sounds, which could suggest a failure to inhibit mismatching speech sounds.

These findings have been replicated in 9‐year‐old children with dyslexia (Blau *et al*., 2010). Specifically, TD children showed significantly more activation in the congruent compared to the incongruent condition, whereas a group of age‐matched children with dyslexia did not. However, as in the adult data, both groups showed a non‐significant increase in activation in the congruent condition compared to speech sounds in isolation. Importantly for the LSI hypothesis, the difference in activation between congruent and incongruent trials in the left hemisphere was moderately correlated with reading accuracy, consistent with the hypothesis that integration is important for efficient decoding.

Two recent studies have investigated letter‐sound congruency effects in other languages. Holloway, van Atteveldt, Blomert and Ansari (2013) conducted an fMRI study of letter‐sound congruency effects in skilled adult readers of English. They did not find a congruency effect in the expected direction for letter‐sound pairs in the STC, which, they suggest, reflects the greater inconsistency of letter‐sound mappings in English. However, if a letter‐sound integration deficit is a putative cause of dyslexia then evidence in support of the hypothesis should be found across a range of alphabetic orthographies, particularly since there are neither orthographic differences in the predictors of reading (e.g. Caravolas, Lervåg, Mousikou, Efrim, Litavský *et al*., 2012; Ziegler, Bertrand, Tóth, Csépe, Reis *et al*., 2010) or in the cognitive deficits observed in children with dyslexia (Landerl, Ramus, Moll, Lyytinen, Leppänen *et al*., 2013). Consistent with this, Richlan (2014) proposes that there is a core dysfunction in dyslexia in all writing systems with additional language‐specific variations. Further, because of this core dysfunction, differences in behavioural or neuroanatomical comparisons across orthographies will be quantitative rather than qualitative (e.g. Paulesu, McCrory, Fazio, Menoncello, Brunswick *et al*., 2000; Paulesu, Démonet, Fazio, McCrory, Chanoine *et al*., 2001). Thus, it is plausible that because the letter sound mappings in English are not consistent, the formation of automatic associations takes longer to develop (a quantitative difference). If this is the case then we would expect the skilled readers in Holloway *et al*.'s (2013) study to show evidence of LSI, given their ample experience.

A study with German‐speaking typically developing and dyslexic adolescents (aged around 16 years) has also produced mixed results. Kronschnabel, Brem, Maurer and Brandeis (2014) collected fMRI and EEG data simultaneously to compare processing of congruent and incongruent CVC and single letter/phoneme pairs. Group differences in the congruency effect were most pronounced in analyses of the CVC stimuli. However, inspection of the fMRI data presented for the single letter/sound condition suggests a lack of a congruency effect in the controls in the left middle temporal gyrus but an effect for the dyslexic group. The EEG scalp maps in the single letter/sound condition appear to be very similar for the two groups. These data are difficult to interpret but suggest that at most there are only minor group differences in LSI for German‐speaking adolescents with dyslexia compared to age‐matched controls. Difficulty replicating automatic LSI effects in typical readers and replicating an absence of automatic LSI in dyslexic readers in other orthographies means that there is a lack of consensus regarding the evidence for the LSI hypothesis and the lack of automatic LSI as a core deficit in dyslexia.

As well as inconsistent findings across different orthographies, there are other limitations of the aforementioned fMRI studies of LSI integration. Specifically, the differences that have been reported between typical readers and those with dyslexia depend upon comparisons between congruent and incongruent conditions. However, during normal reading, a reader does not experience incongruent pairings like those presented in LSI experiments. Moreover, the fact that both dyslexic and typical readers showed a non‐significant increase in fMRI activation for congruent letter‐sound pairs compared to speech sounds in isolation suggests that automatic LSI may be operating normally to some degree in readers with dyslexia. Finally, the lack of reading‐age‐matched controls means that these studies are unable to establish whether differences in automatic integration are a potential cause of reading difficulties or a consequence of reading experience.

In contrast to fMRI studies that have focused on the neural location of the LSI effect, event‐related potential (ERP) studies have explored the strength and timing of LSI using the ‘mismatch negativity’ (MMN) paradigm. The MMN is an auditory ERP component represented by the difference between the brain's response to a frequently presented ‘standard’ sound and a rarely presented ‘deviant’ sound (Näätänen, 2001). When the brain response to the standard is subtracted from the brain response to the deviant, a negative deflection in the difference ERP waveform can be seen at around 200 ms. Froyen, van Atteveldt, Bonte and Blomert (2008) compared an ‘auditory MMN’ (i.e. which used speech sounds for both standard (/a/) and deviant (/o/) stimuli) to a ‘cross‐modal MMN’ (i.e. in which the letter‘a’ was presented with the standard speech sound /a/ or the deviant sound /o/). Compared to the auditory MMN, the cross‐modal MMN produced an enhanced negativity in the ERP waveform to the deviant sound in typical adults. Froyen *et al*. suggested that this was driven by feedback from the processing of the visual letter to the primary auditory cortex, and was interpreted as evidence for early and automatic LSI.

In a later study, Froyen, Bonte, van Atteveldt and Blomert (2009) found that 11‐year‐old children, but not 8‐year‐old children, showed an enhanced MMN in the cross‐modal version compared to the MMN in the auditory version. However, an enhanced late negativity in the ERP waveform (at around 600 ms; sometimes called the ‘late’ MMN) was present in the younger group. These data led the authors to conclude that there is a developmental shift from association (reflected in the late enhanced MMN) to automatic letter‐sound integration (reflected in the early enhanced MMN).

Froyen, Willems and Blomert (2011) went on to show that 11‐year‐old children with dyslexia resembled TD reading‐matched 8‐year‐old children in showing an enhanced late but not early MMN. They attributed the absence of the early MMN in the younger TD group to insufficient reading experience. We propose that the same could be argued of the children with dyslexia; that is, a lack of automatic integration could be a consequence of reading level rather than a cause of reading difficulties. In a recent study, Žarić, González, Tijms, van der Molen, Blomert *et al*. (2014) found that 9‐year‐old typically developing children and 9‐year‐old children with mild dyslexia (as measured by reading fluency) showed an enhanced early cross‐modal MMN, but a group of more severely impaired dyslexic children did not.^1^ Neither dyslexic group showed the enhanced late negativity, which is not consistent with the findings of Froyen *et al*. (2011).

Like the majority of the fMRI data, the MMN data provide some support for the LSI hypothesis. However, there are doubts about the reliability of the MMN component, particularly in children. The auditory MMN is small in amplitude and is not reliably elicited in all individuals. McGee, Kraus and Nicol (1997) tested the success of a variety of methods for detecting real MMNs (wa‐ba stimuli) versus false positives (wa‐wa stimuli) in data from a large sample of children. They found that even the best method still had a high false positive detection rate of 28%. This finding is supported by a number of studies that have found that the MMN is less reliable than other event‐related potential (ERP) peaks, such as the P1, N1, P2 (Badcock, Mousikou, Mahajan, de Lissa, Thie *et al*., 2013; Mahajan & McArthur, 2011; McArthur, Bishop & Proudfoot, 2003). In the current study we chose to focus on the effect of letter‐sound congruency on the auditory ERP waveform, incorporating these peaks.

The aim of the current study was to test the automatic LSI hypothesis using both behavioural and ERP responses in a priming task administered to English‐speaking children with dyslexic difficulties and two groups of controls: a group matched for reading age (RA) who were 7 to 8 years old, and an age‐matched group who were 9 to 12 years old. In the priming task we compared responses to speech sounds (e.g. /t/) preceded by either a congruent visual letter (‘t’) or a neutral letter from the Greek alphabet (‘Ψ’; baseline condition). Given that Holloway *et al*. (2013) did not find the typical automatic LSI effect in English‐speaking adults, it was important to establish whether we could find it in younger typically developing readers at the same time as investigating whether English‐speaking children with dyslexia showed a similar pattern. Using children's behavioural and ERP responses in the congruent and baseline conditions of the priming task, we addressed three aims:

1. To determine whether typically developing (TD) children learning to read in English show evidence of automatic LSI at the level of behaviour or the brain and whether there are developmental differences in this related to age or reading experience.
2. To determine whether children with dyslexia have poor LSI for their age and/or reading level at the level of behaviour or the brain. If poor LSI in children with dyslexia is a genuine deficit, then children with dyslexia should have poor LSI compared to both age‐matched and reading‐matched controls. If it is simply a function of their reading proficiency, then LSI in children with dyslexia should have poorer LSI than age‐matched controls but not reading‐matched controls.
3. To investigate whether behavioural and brain measures of LSI are correlated with reading ability in TD children or children with dyslexia.

## Method

### Ethics statement

Ethical approval for the study was provided by the Ethics Committee of the Department of Psychology at the University of York. Informed written consent was gained from parents of the children who took part in the study. In addition, verbal consent was gained from the children after the study had been explained to them using a child friendly information booklet on the day of testing. Children received a £15 voucher for their participation.

### Procedure

All children in the study completed standardized measures of cognitive skills, a behavioural letter‐sound priming experiment, and a passive (no response required) version of the priming experiment during which EEG data were recorded. After the children had been familiarized with the assessment procedure (using an information booklet), the EEG cap was fitted and then the ERP task was completed. This was followed by a short break. Then the literacy measures and vocabulary subtest were administered. After another short break, the behavioural priming data were collected, and then finally the nonverbal ability task was administered.

### Participants

Forty‐seven children between the ages of 7 and 13 were recruited from an ongoing longitudinal project that compared children at high risk for dyslexia with TD children. The TD children in the current study were either typically developing children from the longitudinal study or their elder siblings, while the children with dyslexic difficulties were the elder siblings of younger at‐risk children in the longitudinal project. There were 13 children in the group with dyslexic difficulties, 17 in a typically developing chronological‐age‐matched group (‘CA’) and 17 in a typically developing reading‐matched group (‘RA’). Ten of the children in the group with dyslexic difficulties had an average standard score of 90 or below on all four standardized tests of literacy (the Single Word Reading Test 6–16 (SWRT6–16); Foster, 2007), the Sight Word Efficiency (SWE) and Phonetic Decoding Efficiency (PDE) subtests from the Test of Word Reading Efficiency (TOWRE; Torgesen, Wagner & Rashotte, 1999) and the Spelling subtest from the Wide Range Achievement Test (WRAT; Wilkinson & Robertson, 2006). The remaining three children had scores below 95. As these children with milder literacy deficits had all been diagnosed as dyslexic by a trained education professional we included them in our sample. However, we ran both the behavioural and EEG analyses with and without these three children so that our results can be compared to previous studies that have used more stringent criteria. We report the analyses including all the children with dyslexic difficulties because excluding them did not affect the results.

### Background cognitive measures

#### Literacy

##### Single word reading

Children completed the Single Word Reading Test 6–16 (SWRT6–16; Foster, 2007) in which they read aloud a list of 60 words that became increasingly difficult. Testing was discontinued after five consecutive errors/refusals.

##### Word reading efficiency

Children completed the Sight Word Efficiency (SWE) and Phonetic Decoding Efficiency (PDE) subtests from the Test of Word Reading Efficiency (TOWRE; Torgesen *et al*., 1999). Children read as many words or nonwords as possible in 45 seconds.

##### Spelling

Spelling was assessed using the subtest from the Wide Range Achievement Test (WRAT 4; Wilkinson & Robertson, 2006). The words gradually increased in difficulty, with a maximum of 42; testing was discontinued after 10 consecutive errors/refusals.

#### General ability

General ability was assessed using two subtests from the Wechsler Abbreviated Scale of Intelligence (WASI; Wechsler, 1999) according to the manual: In Matrix reasoning, children were asked to select the missing design from five alternatives to complete a sequence; for Vocabulary, children were asked to provide a definition for each word read aloud; there were 42 words of increasing difficulty.

Table 1 summarizes information about age, gender and performance on measures of literacy and general ability for each group. The dyslexic group was similar in age to the CA group and equated in reading and spelling levels to the RA group. Despite being matched for word reading, the dyslexic group did significantly less well on the timed test of decoding than the RA control group. All three groups had mean scaled scores on the general ability tests that were within the normal range and did not differ significantly.

**Table 1:** Mean (*SD*) age and scores on standardized measures of literacy and general cognitive ability in the three groups

	Dyslexic (*N* = 13)	CA (*N* = 17)	RA (*N* = 17)	Significance testing	Group differences
Gender (% male)	62%	53%	59%	*χ* ^2^ = 24, *p = *.885	none
Age (months)	137.00 (19.60)	128.00 (14.46)	100.88 (6.63)	*F *= 28.04, *p* = .000	(DYS = CA) > RA
SWRT raw score	36.69 (11.78)	50.06 (6.17)	39.12 (5.51)	*F *= 12.79, *p *= .000	CA > (DYS = RA)
SWRT standard score^1^	85.92 (10.94)	110.35 (12.54)	103.00 (9.23)	*F *= 18.66, *p* = .000	(CA=RA) > DYS
TOWRE SWE raw	57.23 (16.08)	75.59 (9.71)	60.71 (10.63)	*F *= 10.29, *p* = .000	CA > (DYS = RA)
TOWRE SWE standard^1^	88.61 (11.11)	110.65 (10.72)	110.88 (10.95)	*F *= 19.37, *p* = .000	(CA = RA) > DYS
TOWRE PDE raw	20.23 (8.94)	43.82 (11.20)	30.06 (6.85)	*F *= 25.07, *p* = .000	CA > RA > DYS
TOWRE PDE standard^1^	83.77 (7.89)	113.65 (14.39)	108.24 (9.24)	*F *= 29.17, *p* = .000	(CA = RA) > DYS
WRAT raw	26.77 (4.09)	36.59 (6.66)	26.47 (4.45)	*F *= 19.43, *p* = .000	CA > (DYS = RA)
WRAT standard^1^	86.77 (8.71)	114.06 (16.22)	105.00 (15.35)	*F *= 13.78, *p* = .000	(CA = RA) > DYS
WASI vocabulary raw	39.71 (8.94)	44.76 (8.06)	32.65 (7.47)	*F *= 9.45, *p* = .000	CA > RA
WASI vocabulary *t*‐score^2^	50.21 (9.18)	58.41 (9.95)	55.41 (11.34)	*F *= 2.84, *p* = .069	none
WASI matrices raw	24.07 (6.21)	25.29 (4.10)	21.18 (4.85)	*F *= 2.86, *p* = .068	none
WASI matrices *t*‐score^2^	54.21 (9.09)	57.29 (7.34)	59.35 (7.36)	*F *= 2.63, *p* = .084	none

Notes

^1^Standard score with a mean of 100 and a standard deviation of 15. ^2^ *T*‐score with a mean of 50 and a standard deviation of 10.

### Behavioural priming measure

Within the priming task there were four conditions: congruent, baseline and two control conditions. In each condition the child's task was to decide whether the speech sound was a ‘real sound’ or not (see Table 2 for example trials in each condition). In the congruent condition, the prime and target were congruent letters and sounds (‘t’ → /t/). In the baseline condition, the neutral prime was a Greek letter and the target was a speech sound (‘Ψ’ → /t/). A faster response to a speech sound presented after a congruent letter in comparison to a neutral Greek letter reflects facilitation caused by the congruent letter. The two control conditions were included simply to prevent children generating expectancies about the up‐coming target and to balance the yes/no responses to the speech sound. Thus, the data from the control conditions were not analysed. In the first control condition the prime was a real letter and the target was a scrambled phoneme (‘t’ → /t^s^/). In the second control condition the prime was a Greek letter and the target was a scrambled phoneme (‘Ψ’ → /t^s^/). The same Greek letter was always presented in the baseline trials for each English letter; these pairings were: t‐Ψ, d‐Ω, k‐λ, v‐π, j‐φ, p‐Ξ, z‐δ.

**Table 2:** Example trials for each condition of the priming task

Condition	Prime	Target	Response: Real letter sound?
Congruent	t	/t/	YES
Baseline	Ψ	/t/	YES
Control 1	t	/t^s^/	NO
Control 2	Ψ	/t^s^/	NO

Phonemes were: /p/ (283 ms), /t/ (293 ms), /d/ (263 ms), /k/ (304 ms), /v/ (428 ms), /z/ (413 ms) and /j/ (357 ms). The phonemes were recorded by a female native English speaker in a sound attenuated booth. Speech sounds were scrambled using Matlab (2010). Each sound was divided into 5 ms overlapping Hanning windows. The order of these windows was then randomized within a 250 ms radius. The randomly overlapping windows were then combined to form the scrambled speech sound. The length, overall power and frequency spectrum remained as similar as possible to the original phonemes. Greek letters were used for non‐letter stimuli (Ψ, Ω, λ, π, φ, Ξ, δ) as they are visually complex letter forms but unlikely to be familiar to the children in the study.

The priming task was run using Eprime version 2 (Schneider, Eschmann & Zuccolotto, 2002). At the beginning of the task, children were tested on their knowledge of the seven English letters used. They were asked to produce the sound for each one. All the children could do this. This was followed by practice trials. First, each auditory stimulus was presented in isolation and the child was required to respond using yes/no keys as to whether each was a real speech sound or not. Trials were repeated if the child responded incorrectly. Next the prime was introduced and children completed a further nine practice trials (two from the related condition, two from the unrelated condition, four from the control conditions and a catch trial). Catch trials were included to ensure that the children were attending to the visual primes. Catch trials consisted of a colourful picture of a present, to which the child was required to press the spacebar to collect points towards their reward for taking part. These practice trials were run in a fixed, pseudo‐randomized, order. Children were instructed to attend to the visually presented letter, listen to the auditorially presented target, and then respond to that target.

There were 56 experimental trials, two of each pairing in each condition (14 trials in each condition), and eight catch trials to ensure that the children were attending to the stimuli. Prior to each trial a visually presented ‘Ready?’ stimulus appeared on the screen. When the child was ready the experimenter clicked the mouse to start the trial. In each trial a central fixation cross was presented for 1000 ms to guide the participant's attention. Next a black visual prime appeared on a white screen for 500 ms. The letters were written in Arial font size 56. They were presented on a 17 inch monitor with a screen resolution of 1280 × 1024 and the children sat 50 cm from the screen. Although there was some variation for individual letters, the approximate average size of the stimuli in pixels was 1 inch square. With a PPI of 96.88, the horizontal visual angle was 3.58 and the vertical was 3.90. The visual letter was followed by another fixation cross which remained on the screen for the ISI of 500 ms. The auditory target was then presented over headphones at a comfortable listening level followed by the visually presented ‘Real sound?’ probe which prompted the participant to make a response. Accuracy and response time were recorded for each trial.

### ERP priming measure

A slightly modified version of the priming task was run using Presentation^®^ software (www.neurobs.com) while each child's EEG was recorded for the ERP responses. This differed from the behavioural priming task in only two ways. First, children were not required to make physical responses to the stimuli. Instead, they were asked to look at the letters on the computer screen and listen to the sounds. Catch trials were included to maintain their attention. Second, to maximize the reliability of the ERP data, the ERP priming task used 308 experimental trials (rather than 56) and 30 catch trials (rather than 8). There were 11 trials of each of the seven letter‐sound pairings, in each of the four conditions, resulting in 77 trials per condition. The experiment took approximately 12 minutes to complete. The children were given a break after the first 6 minutes

### EEG recording and ERP processing

We recorded children's EEGs from 30 electrodes mounted on a cap with shielded wires (Waveguard active shield cap, Advanced Neuro Technology system, ANT) that were positioned according to the Extended International 10–20 System (American Electroencephalographic Society, 1994) (Fp1, Fpz, Fp2, F7, F3, Fz, F4, F8, FC5, FC1, FC2, FC6, T7, C3, Cz, C4, T8, CP5, CP1, CP2, CP6, P7, P3, Pz, P4, P8, POz, O1, Oz, O2). In addition, four bipolar electrodes were used to record vertical and horizontal eye movements. Data were recorded with a common average reference and signals were amplified using a full‐band EEG DC amplifier. The data were sampled at 1000 Hz and online filtered with a 0.5 Hz high‐pass and 500 Hz low‐pass using the ASA system. Electrode impedance levels were kept below 4 kΩ.

The raw EEG data were processed offline using EEGLAB v12.0.2.2 (Delorme & Makeig, 2004). The EEG from each electrode was re‐referenced to the average of the 30 scalp electrodes, down‐sampled to 250 Hz, and filtered with a 1 Hz high‐pass and a 30 Hz low‐pass filter. An independent component analysis (ICA) was run on the filtered data in order to reduce artefacts caused by eye blinks, eye movements, and muscular activity. Following this, each EEG trace was divided into 500 ms epochs that began 200 ms before the onset of a stimulus and ended 300 ms after the onset of the same stimulus. After excluding the four non‐scalp electrodes, single epochs were rejected if amplitudes exceeded ±100 μV, if linear trends exceeded 100 μV in a 200 ms gliding window, or if the trial was lying outside a ± 5 *SD* range (for a single channel) or ±3 *SD* range (for all channels) of the mean probability distribution or the mean distribution of kurtosis values. Following data processing, two children in the younger RA group and one child in the older CA group were excluded from further analysis due to excessive artefacts. The pre‐processed data were finally baseline‐corrected in the pre‐stimulus window ranging from −200 to 0 ms. The mean number of epochs in each condition, in each of the three groups, is shown in Table 3. For each participant we then averaged together all their accepted EEG epochs in congruent trials to form their ‘congruent ERP’, and we averaged together all their accepted epochs in baseline trials to produce their ‘baseline ERP’.

**Table 3:** Mean number of epochs (*SD*) in the baseline and congruent conditions in each group

	CA (*N* = 16)	RA (*N* = 15)	DYS (*N* = 13)
Baseline	53.81 (8.48)	47.67 (9.18)	47.15 (9.86)
Congruent	53.25 (6.03)	48.80 (8.01)	49.08 (9.58)

## Results

We will present the behavioural data from the priming task first, before proceeding to the ERP data. Finally we will consider correlations between the behavioural and ERP measures of LSI and reading accuracy.

### Behavioural priming data

**Figure 1:**
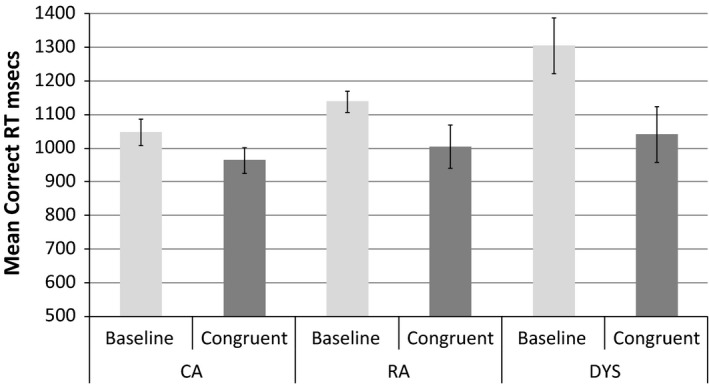
Mean RTs, with 95% within‐subjects confidence intervals, in the baseline and congruent conditions in the dyslexic, CA and RA groups.

The children found this task quite straightforward and accuracy levels were high. All three groups achieved near perfect levels of performance (max = 14, mean accuracy >13 in both conditions in all three groups). Before analysing reaction times (RTs) for correct responses, we removed outliers, defined as values greater than 2.5 *SD*s above or below an individual child's mean RT in that condition (as recommended by Ratcliff, 1993). The total percentage of RT data excluded, as both response errors and outliers, ranged from 2 to 7% across the conditions and groups. Thus over 90% of the possible RT data were available for analysis, in each condition, in each group. The mean RTs in the two conditions, in each group, are shown in Figure 1.

In the baseline condition, where the prime was a neutral Greek letter, there was a clear step pattern in the data: the CA group had faster RTs than the RA group who in turn were faster than the dyslexic group. The RTs in the congruent condition were similar across the groups. Crucially, all three groups showed evidence of priming, with shorter RTs in the congruent condition than in the baseline. Thus, presentation of a visual letter facilitated subsequent processing of the corresponding speech sound.

We performed a 3 (group) × 2 (prime type) mixed ANOVA to test whether there were significant main effects of congruency or group on reaction times and whether there was a significant interaction (i.e. differences in priming between the three groups). The criterion for statistical significance was *p *<* *.05. There was a significant main effect of condition (*F*(1, 44) = 31.63, *p *=* *.00, $\eta_{p}^{2}$ = .42) but no significant effect of group (*F*(2, 44) = 1.06, *p *=* *.36, $\eta_{p}^{2}$ = .05) or significant group × condition interaction (*F*(2, 44) = 2.97, *p *=* *.06, $\eta_{p}^{2}$ = .12). This analysis supports the observation above; in all three groups and to a statistically similar degree, behavioural responses to the speech sounds were facilitated in the congruent condition.

### ERP priming data

**Figure 2:**
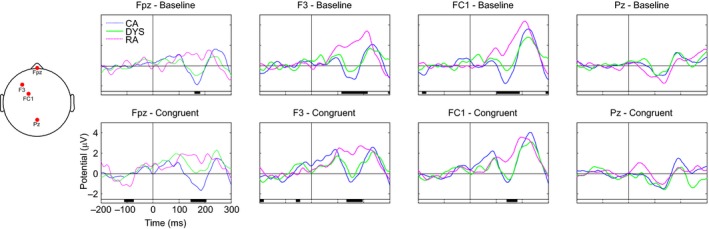
Event‐related brain potentials (ERPs) for the between‐groups comparison at four selected electrodes (Fpz, F3, FC1, and Pz). The three groups of children (age‐matched ‘CA’, reading‐matched ‘RA’, and dyslexic ‘DYS’) are compared against each other in the two conditions (‘baseline’ and ‘congruent’). The black bar under each panel indicates times at which there was a significant difference between the three groups. Statistical differences were assessed with permutation tests (*n *=* *2000) corrected for multiple comparisons (Holms method) and an alpha level of 0.05.

**Figure 3:**
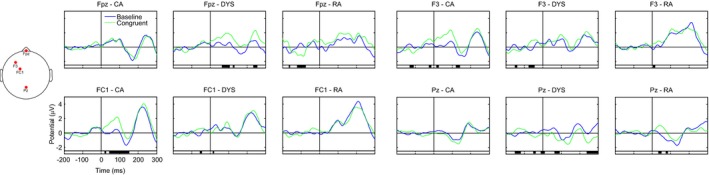
Event‐related brain potentials (ERPs) for the within‐groups comparison at four selected electrodes (Fpz, F3, FC1, and Pz). The two conditions (‘baseline’ and ‘congruent’) are compared against each other in the three groups of children (age‐matched ‘CA’, reading‐matched ‘RA’, and dyslexic ‘DYS’). The black bar under each panel indicates times at which there was a significant difference between the two conditions. Statistical differences were assessed with permutation tests (*n *=* *2000) corrected for multiple comparisons (Holms method) and an alpha level of 0.05.

We ran a permutation test (*n *=* *2000) implemented in EEGLAB on the cleaned data from the 16 CA, 15 RA and 13 dyslexic children, with corrections for multiple comparisons (Holms method) and an alpha level of 0.05. The permutation test has been shown to be valid for dealing with the problem of multiple comparisons in the ERP analysis, avoiding the ad hoc selection of channels and/or time windows (Lage‐Castellanas, Martinez‐Montes, Hernández‐Cabrera & Galán, 2010). This method tests for differences across the whole epoch length (−200–300 ms) and across the entire array of electrodes. It also allows for differences both between groups (effects of age and reading level) and within groups (effect of congruency) to be tested. Figures 2 and 3 show the ERP waveforms for four representative electrodes (Fpz, F3, FC1, Pz) at which we observed the largest effects between the groups in Figure 2 and within each group in Figure 3. In Figure 2 the ERPs from each group are plotted in the same panel, in the baseline condition (top row) and the congruent condition (bottom row). The black bar underneath each panel indicates the timing of significant differences between the baseline and congruent conditions in each of the three groups. In Figure 3 the waveforms from the two conditions in each group are plotted in the same panel at each electrode site. The black bar underneath each panel indicates the timing of significant differences between the two conditions (congruency effect) within a group.^2^

**Figure 4:**
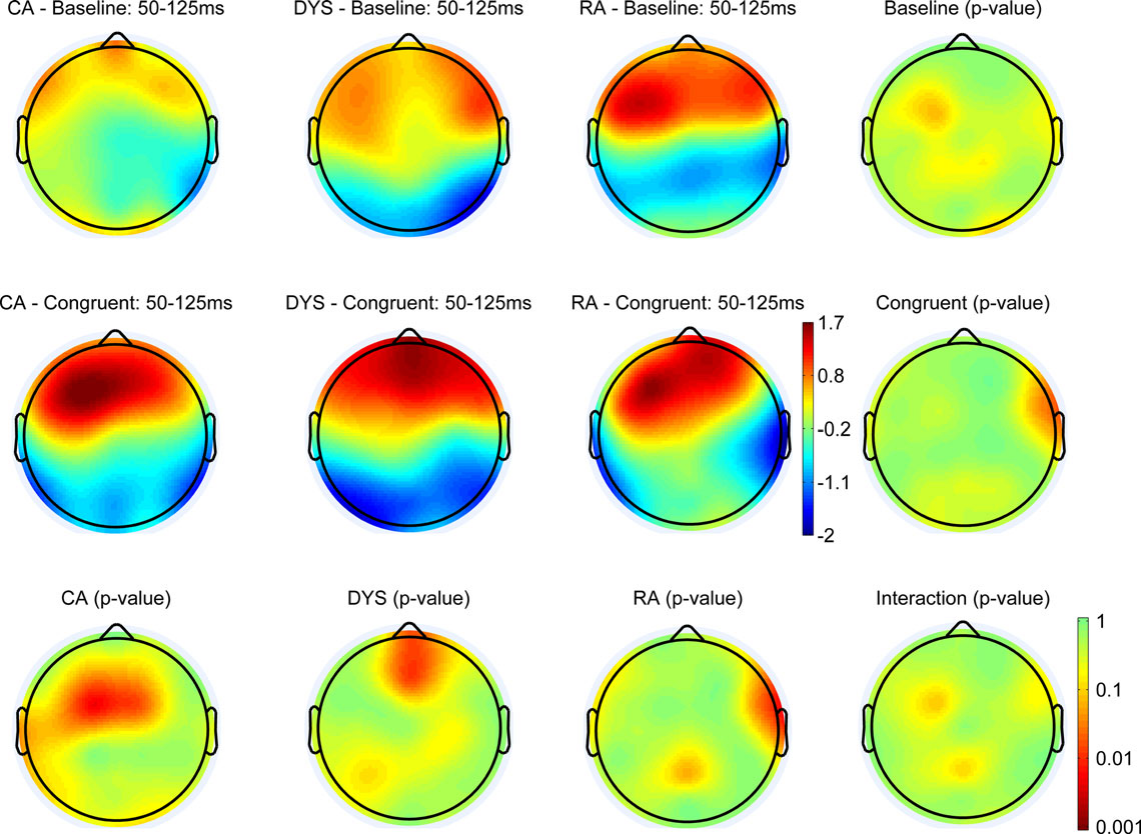
Scalp distributions of the ERPs in each of the three groups and two conditions between 50 and 125 ms. The right column and bottom row show p‐value distributions for the statistical comparisons between and within groups. Statistical differences were assessed with permutation tests (n = 2000) corrected for multiple comparisons (Holms method).

**Figure 5:**
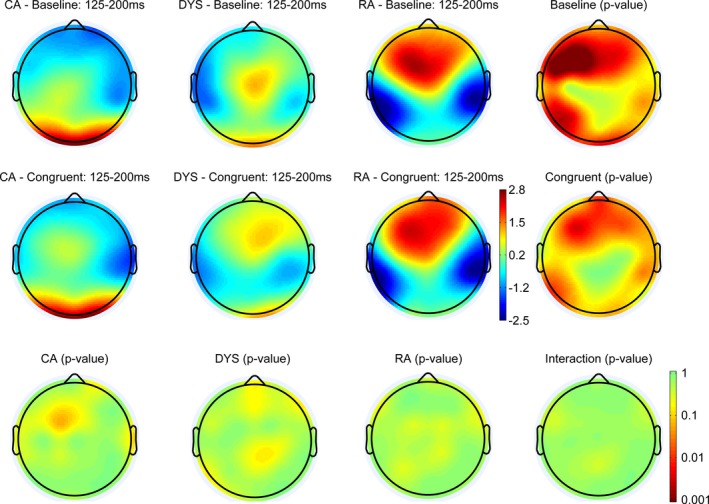
Scalp distributions of the ERPs in each of the three groups and two conditions between 125 and 200 ms. The right column and bottom row show *p*‐value distributions for the statistical comparisons between‐ and within‐groups. Statistical differences were assessed with permutation tests (*n *=* *2000) corrected for multiple comparisons (Holms method).

**Figure 6:**
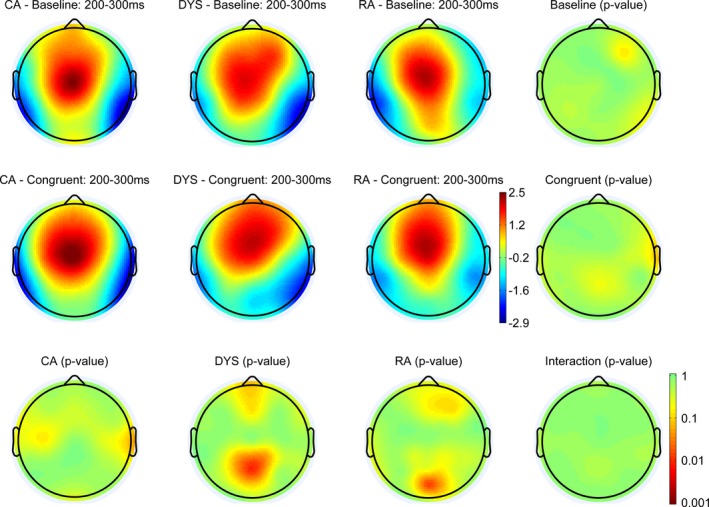
Scalp distributions of the ERPs in each of the three groups and two conditions between 200 and 300 ms. The right column and bottom row show *p*‐value distributions for the statistical comparisons between and within groups. Statistical differences were assessed with permutation tests (*n *=* *2000) corrected for multiple comparisons (Holms method).

Figures 4, 5 and 6 show the scalp maps for three auditory ERP components, P1 (50–125 ms), N1 (125–200 ms), P2 (200–300 ms), respectively. We have depicted relatively long windows to take account of any potential differences in latency. The top row shows the scalp distribution of the ERPs in the baseline condition, the middle row shows the distribution of ERPs in the congruent condition and the bottom row shows any significant differences between these two conditions. The scalp distributions of the CA group are shown in the left‐hand column, with those of the dyslexic group in the next column to the right and those of the RA group in the next column along to the right. The furthest right column shows any significant differences in each condition, between the three groups.

#### P1 (50–125 ms)

All three groups showed a P1 peak in the time window. The scalp maps show that in the CA and RA groups, but not the dyslexic group, activation was more left‐lateralized in the congruent compared to the baseline condition. Significant differences in amplitude between the two conditions (congruency effect) were evident for the CA and dyslexic groups, with greater amplitude in the congruent condition. In the CA group the effect was most pronounced at fronto‐central electrodes and left‐lateralized, whereas the difference had a more central and pre‐frontal scalp distribution in the dyslexic group. A significant effect was also evident in the RA group. However, rather than being driven by a difference in the central region, where activity was strongest in both conditions, it appears to reflect activation more strongly left‐lateralized in the congruent condition. This speaks against it being a genuine effect of condition on congruency as seen in the CA and dyslexic groups.

#### N1 (125–200 ms)

Within the N1 window there was evidence of a between‐groups difference. Inspection of the ERP waveforms revealed that this was driven by a lack of an N1 in the RA group. There was no evidence of a congruency effect in any of the groups in the N1 time window.

#### P2 (200–300 ms)

All three groups showed evidence of a P2 peak in this time window and there were no significant differences between them. Both the dyslexic and RA groups showed a significant effect of congruency in the central posterior region, with greater amplitude in the baseline condition.

### Relationships between the measures of LSI and reading accuracy

We computed partial correlations (controlling for chronological age) between a composite of reading accuracy (summed raw scores from the SWRT and the two TOWRE subtests) and congruency effects (i.e. difference between responses in the congruent and baseline conditions) in behavioural priming responses (i.e. RTs) and ERP waveforms. We present analyses separately for the CA and dyslexic groups combined and the RA and dyslexic groups combined, due to the different congruency effects seen in these groups in the ERP data.

Within the combined CA and dyslexic sample the correlation between the behavioural index of congruency and reading accuracy was moderate and negative but non‐significant (*r *= −.25, *p *>* *.20), which suggests that the poorer readers showed larger behavioural priming effects. In the CA and dyslexic groups, we measured the peak P1 amplitude in the baseline and congruent conditions within the 50–125 ms time window at two electrode sites for each participant. The choice of electrodes was determined by the results of the permutation test and can be seen in Figure 3. In the CA group the largest P1 congruency effect was at FC1, while in the dyslexic group it was at Fpz. For each participant we calculated the difference in peak amplitude between the baseline and congruent conditions, so that positive values reflected greater congruency effects. There was a moderate, but non‐significant correlation between the effect of congruency on P1 at FC1 and reading accuracy (*r *=* *.27, *p *>* *.17) and between the effect of congruency on P1 at Fpz and reading accuracy (*r *= −.23, *p *>* *.25). The relationship between the congruency effect at FC1 and reading accuracy was positive, a larger congruency effect was associated with higher reading accuracy. Whereas, the relationship between the congruency effect at Fpz and reading accuracy was negative, a larger congruency effect was associated with lower reading accuracy.

When the dyslexic and RA groups were combined, the correlation between behavioural priming and reading accuracy was small and non‐significant (*r *=* *.12, *p *>* *.56). In the CA and dyslexic groups, we measured the peak P2 amplitude in the baseline and congruent conditions within the 50–125 ms time window at Pz for each participant. The correlation between the effect of congruency on P2 and reading accuracy was small and non‐significant (*r *=* *.16, *p *>* *.41).

## Discussion

The aim of the current study was to test the LSI hypothesis (Blomert, 2011), which posits that the proximal cause of dyslexia is a failure to adequately integrate letters and speech sounds into fully automated audio‐visual objects. To this end, we investigated whether children with dyslexic difficulties, chronological‐age and reading‐age‐matched controls showed evidence of LSI in their behavioural and ERP responses in a priming paradigm. We had three main aims at the outset and we will discuss the findings relating to each of these in turn.

### Do typically developing children learning to read in English show evidence of automatic LSI and is this related to age/reading experience?

At the outset of this study we argued that if a letter‐sound integration deficit is a putative cause of dyslexia then evidence in support of the hypothesis should be found across a range of alphabetic orthographies. However, given that English is a more opaque orthography, we hypothesized that the formation of automatic letter‐sound associations may take longer to develop than in more transparent orthographies. The authors of the Dutch MMN studies interpreted their findings as showing a developmental shift from association seen in typically developing 8‐year‐olds (late MMN effect) to automatic integration in 11‐year‐olds (early MMN effect). The typically developing children in our study were divided into two groups, similar in age to the groups in the Dutch studies. Both groups showed a congruency effect on the behavioural priming task. That is, responses to the speech sounds were facilitated by the presentation of congruent letters compared to neutral Greek letters. From the perspective of van Atteveldt *et al*. (2004), the letter prime in our task would have activated the visual cortex and then the heteromodal STS and STG areas. This activation would have fed back to primary auditory cortex and activated the corresponding speech sound. The key question is whether the facilitative effect of the letter on the speech sound reflects automatic integration or whether priming could have been driven by letter‐sound associations that were not fully automatized.

The ERP data can be used to address this question since a particular strength of ERPs is that they reflect the time course of cognitive processing. In contrast to the behavioural data, developmental differences were evident. In the older CA group, the amplitude of the P1 component (50–125 ms after onset) was significantly greater over left, fronto‐central electrodes when the speech sound was preceded by a congruent visual letter. This congruency effect was not evident in the younger, RA controls, although in both age groups activation was more left‐lateralized in the congruent condition. The P1 is usually interpreted as a neurophysiological indicator of preferential attention to sensory input (Key, Dove & Maguire, 2005). This suggests that the larger P1 amplitude following a congruent visual letter reflects cognitive processing at an early sensory level. In the younger RA group a congruency effect was observed later, at approximately 200–300 ms within the P2 time window, with greater amplitude in the baseline condition over central parietal electrodes. P2 often occurs together with N1 in the auditory modality and it shares many characteristics of the preceding component but can be dissociated. P2 amplitude is sensitive to attention and stimulus intensity, which suggests that the congruency effect may reflect attentional processing.

The age‐related differences in our ERP data mirror those found in the MMN studies by Froyen *et al*. (2009, 2011), in which Dutch children aged 11 years showed an early enhanced cross‐modal MMN while 8‐year‐olds showed a later enhanced negativity. These data led the authors to conclude that these reflect a developmental shift from letter‐sound association to automatic letter‐sound integration. Our data could be interpreted in a similar way. Specifically, the P1 congruency effect might reflect automatic LSI while the P2 might reflect association. An alternative explanation is that both the P1 and P2 congruency effects are driven by automatic integration, but that automatic activation of speech sounds from letters takes longer in less experienced readers, hence the effect becomes evident later in the ERP waveform. With increased reading experience, associations between letters and sounds are strengthened, resulting in more efficient neural networks that can detect congruency more quickly, and hence effects emerge earlier in the ERP waveform. However, in both cases, when a visual letter is presented, activation of the heteromodal areas in the ST and STG feeds back to primary auditory cortex and activates the corresponding speech sound. The results of this study suggest that the behavioural priming task is not as sensitive to the timing of this feedback and so both groups showed facilitation.

In our earlier discussion of how automatic LSI might develop in children learning to read English, we hypothesized that automatic letter‐sound associations would take longer to develop due to the inconsistency of the mappings (i.e. more reading experience). On first inspection the developmental pattern in our data suggests this is not the case since the two groups of children in our study were of a similar age to those in the Dutch MMN studies. However, it should be noted that formal literacy instruction begins at a younger age in the UK, thus the children in our sample will have had more reading experience than their Dutch counterparts. In order to test the hypothesis that the time course of the development of automatic associations varies with orthographic transparency, a cross‐linguistic study comparing children with similar amounts of reading experience on the same LSI task would be necessary.

An advantage of the current study was the inclusion of the age‐ and reading‐age‐matched comparison groups. In doing this we were able to investigate LSI across development and compare dyslexic readers to children from each group to determine whether they show abnormal LSI for their age and/or reading age. However, we acknowledge that auditory ERPs mature with age and we anticipated that we might see age‐related differences between the groups. In line with previous literature (e.g. Pang & Taylor, 2000), we did see a reduced N1 (in both conditions) in the younger controls. However, no such developmental differences were evident for the P1 or P2 components. Thus, we propose that differences in the timing of the congruency effect (50–125 ms in the older group compared to 200–300 ms in the younger group) reflect underlying differences in either the speed of automatic integration or in the nature of associations between letters and sounds in the two groups rather than age‐related differences in the ERP waveforms.

### Do children with dyslexia show a genuine deficit in LSI integration or is their performance a function of their reading ability?

Like both typically developing groups, the children with dyslexic difficulties showed a congruency effect in the behavioural priming data. However, given that we found developmental differences in the ERP data, this priming effect may not reflect automatic integration. Indeed, the ERP data presented a more complex picture, in which the children with dyslexic difficulties showed similarities with older and younger controls, providing evidence for both an early and a late congruency effect. While the late P2 congruency effect was indistinguishable from that of the younger control group, the early P1 effect was not found across the same electrodes as in the older control group. The significant effect of congruency on P1 was more frontal and centrally located in the children with dyslexic difficulties, suggesting that the effect in the two groups might have a different underlying source. Furthermore, in both typically developing groups activation was more left‐lateralized in the congruent compared to the baseline condition but this was not evident in the children with dyslexia. Greater amplitude over the frontal region in the congruent condition may reflect greater processing effort in this condition in the children with dyslexic difficulties (fMRI studies have shown that higher cognitive loads results increased level of BOLD activity in the pre‐frontal cortex e.g. Sabri, Humphries, Verber, Liebenthal, Binder *et al*., 2014).

Previous studies with dyslexic readers in other orthographies have not found evidence of automatic letter‐sound integration. Our aim was to further this research using an alternative methodology and a reading‐age‐matched control group. We argue that in doing this we have been able to show a degree of integration that is in line with reading ability. Another potential difference with previous studies concerns our sample. It could be argued that our sample contains children who are less severely affected than the children in other samples. Dyslexia is now accepted to represent a continuum of impairment (Peterson & Pennington, 2012); however, for research purposes, a cut off has to be set at some point along this continuum. Our research criterion of an average standard score < 90 could be seen as fairly lenient. However, this is an average across four widely used literacy measures; word and nonword reading accuracy, reading fluency and spelling, and the children's scores on these measures did in fact range from severe to moderate (in the sample of 10) and mild (in the full sample of 13). When we re‐ran our analyses excluding the three mildly impaired children, the results were the same. Indeed, the majority of the remaining 10 children have an average standard score below 85 and nearly all have a nonword reading score below 85. Finally, all the children in our dyslexic sample have received a diagnosis of dyslexia from an education professional; we cannot know what impact subsequent support has had on their literacy levels.

### Are variations in performance on measures of automatic LSI correlated with decoding scores?

According to the LSI hypothesis, automatic integration is required for retrieving speech sounds from letters during reading to enable efficient decoding. Conversely, experience learning letter sounds and letter‐by‐letter decoding is the means by which letter and speech sound representations become integrated. Therefore, we would expect the relationship between LSI and decoding to be bi‐directional in nature, and hence measures of the two constructs to correlate significantly. In their fMRI study, Blau *et al*. (2010) found that in a sample of typically developing children and children with dyslexia, the difference in activation between congruent and incongruent trials in the left hemisphere was moderately correlated with reading accuracy. In our study we tested whether our behavioural and ERP indices of LSI were concurrently related to reading accuracy. The correlation between the left‐lateralized early congruency effect and reading was in the expected direction and moderate in strength; better readers showed a greater difference between P1 amplitude in the congruent and baseline conditions. However, contrary to what would be expected if a frontally located effect reflects automatic LSI, the moderate correlation between the frontal early congruency effect and reading was in the opposite direction; poorer readers showed the greatest difference, suggesting that letter‐sound integration is more effortful for the poorest readers. In line with this, the correlation between the behavioural measure of priming and reading was also negative in the older and dyslexic groups. In this task the poorest readers showed the greatest facilitation of the congruent letter on speech sound processing. The dyslexic readers certainly had slower reaction times in the baseline condition, suggesting that they were slower to decide whether the speech sound was real or not. These readers may have paid greater attention to the letter primes to aid their performance, whereas the typical readers, who were already responding quickly, did not need to allocate as much attention to the prime to enable them to complete the task. This suggests that the behavioural priming task may not be a pure measure of automatic LSI, which is in line with the lack of developmental differences on this task. The later P2 congruency effect was only weakly correlated with reading, which again could be taken to suggest that this effect reflects association rather than the automatic LSI that is implicated in efficient reading.

We can summarize the key findings of the current study as follows. In the ERP data collected during our letter‐sound priming task we found evidence of a developmental shift from a late to an early congruency effect on amplitude. This is in keeping with the developmental shift seen in the MMN task in studies of Dutch children. The developmental differences picked up in the EEG task lead us to propose that the behavioural priming effect observed in both the older and younger typical readers may be driven by either automatic LSI or letter‐sound associations. Our sample of children with dyslexic difficulties demonstrated a degree of letter‐sound integration or association that was appropriate for their reading level, in the form of the later congruency effect on P2. It could be argued that they did in fact show evidence of early, automatic LSI that is more in line with their chronological age. However, the P1 congruency effect in the children with dyslexic difficulties was more frontal and we have speculated that it may reflect processing effort rather than automatic integration. However, because of the relatively poor spatial resolution of EEG, we urge a degree of caution in attributing the frontal P1 seen in the children with dyslexic difficulties to a different underlying source compared to the left P1 in the older typical readers. While we acknowledge that replication is important, the current findings suggest that reading experience may determine the degree to which children are able to integrate letters and speech sounds. It is possible that with more reading experience the children with dyslexic difficulties would eventually show the left‐lateralized P1 congruency effect seen in the more advanced typical readers but longitudinal data are needed to answer this question.

## Footnotes

1 The main effect of group was not significant in a mixed ANOVA, but pairwise group comparisons showed that there was a significant difference between the severely dysfluent dyslexics and typical readers.

2 We note that there are some significant differences during the baseline period; however, the majority of the waveforms are aligned at 0 ms.
